# Sal B Alleviates Myocardial Ischemic Injury by Inhibiting TLR4 and the Priming Phase of NLRP3 Inflammasome

**DOI:** 10.3390/molecules24234416

**Published:** 2019-12-03

**Authors:** Yang Hu, Qingju Li, Yunzheng Pan, Li Xu

**Affiliations:** Jiangsu Key Laboratory for Pharmacology and Safety Evaluation of Chinese Materia Medica, School of Pharmacy, Nanjing University of Chinese Medicine, Nanjing 210023, China; 20171461@njucm.edu.cn (Y.H.);

**Keywords:** Sal B, priming phase, TLR4, NF-κB, NLRP3 inflammasome

## Abstract

Salvianolic acid B is one of the main water-soluble components of Salvia miltiorrhiza Bge. Many reports have shown that it has significant anti-myocardial ischemia effect. However, the underlying mechanism remains unclear. Our present study demonstrated that Sal B could alleviate myocardial ischemic injury by inhibiting the priming phase of NLRP3 inflammasome. In vivo, serum c-troponin I (cTn), lactate dehydrogenase (LDH) levels, the cardiac function and infract size were examined. We found that Sal B could notably reduce the myocardial ischemic injury caused by ligation of the left anterior descending coronary artery. In vitro, Sal B down-regulated the TLR4/NF-κB signaling cascades in lipopolysaccharide (LPS)-stimulated H9C2 cells. Furthermore, Sal B reduced the expression levels of IL-1β and NLRP3 inflammasome in a dose-dependent manner. In short, our study provided evidence that Sal B could attenuate myocardial ischemic injury via inhibition of TLR4/NF-κB/NLRP3 signaling pathway. And in an upstream level, MD-2 may be the potential target.

## 1. Introduction

Data showed that cardiovascular disease (CVD) is the main cause of death nowadays [1]. Myocardial ischemia plays an important role in it. Severe myocardial ischemia could lead to heart failure, myocardial infarction and other harmful consequences. Although a lot of studies have been carried out into myocardial ischemia, the molecular mechanisms involved in the occurrence and development of myocardial ischemia injury are still not fully understood yet. Therefore, we urgently need to understand the myocardial ischemia injury mechanism and find appropriate strategies to deal with it.

Inflammation is one of the main causes of myocardial ischemic injury. Many reports have shown that the reducing of the inflammatory response helps to alleviate myocardial ischemic injury [2,3,4]. TLR4/NF-κB signaling pathway is closely related to inflammation. When the body is stimulated by different factors, TLR4 is over-activated, which then activates Myd88-dependent pathway and finally activates NF-κB, leading to a series of inflammatory cascades. Inhibiting the activation of TLR4/NF-κB signaling pathway contributes to reducing the expression of many pro-inflammatory cytokines and mitigating myocardial ischemia [5,6,7]. Besides, the NOD-, LRR- and pyrin domain-containing protein 3, known as NLPR3, which was observed increased expressed and expand inflammatory response, was associated with the pathogenesis of many inflammatory diseases and regulated the secretion of many pro-inflammatory cytokines [8]. 

The activation of classical NLRP3 inflammasome requires the involvement of both priming phase and triggering phase. The priming phase is a process which leads to the activation of NF-κB and the transcription of inflammasome components. The triggering phase refers to the activation and the assembly of NLRP3 inflammasome. Studies have shown that short-term treatment with LPS in macrophages can directly activate NLRP3 inflammasome bypassing the priming phase [9]. However, the priming phase and triggering phase are two equally important mechanisms for the formation of NLRP3 inflammasome in the heart. Prevention of NLRP3 activation in the priming phase during acute myocardial ischemia is sufficient to inhibit the inflammasome and protecting heart function [10].

Salvianolic acid B (Sal B) is a phenolic acid isolated from Salvia miltiorrhiza Bge. It has been shown to have a variety of pharmacological activities, such as antioxidant [11], anti-myocardial ischemia [12], anti-tumor [13], anti-inflammatory activities [14]. Previous studies have shown that Sal B has a good anti-myocardial ischemic effect [15,16]. However, whether Sal B could reduce myocardial ischemia injury by inhibiting the activation of NLRP3 inflammasome remains unknown. We hypothesize that the mechanism of salvianolic acid B against myocardial ischemia may be related to the inflammatory cascade induced by TLR4/NF-κB/NLRP3 signaling pathway. Therefore, the present study was designed to investigate the anti-myocardial ischemic effect of Sal B and explore its underlying mechanism.

## 2. Results

### 2.1. Effect of Sal B on Myocardial Infarct Size in Myocardial Ischemia Injury Rats

**Figure 1:**
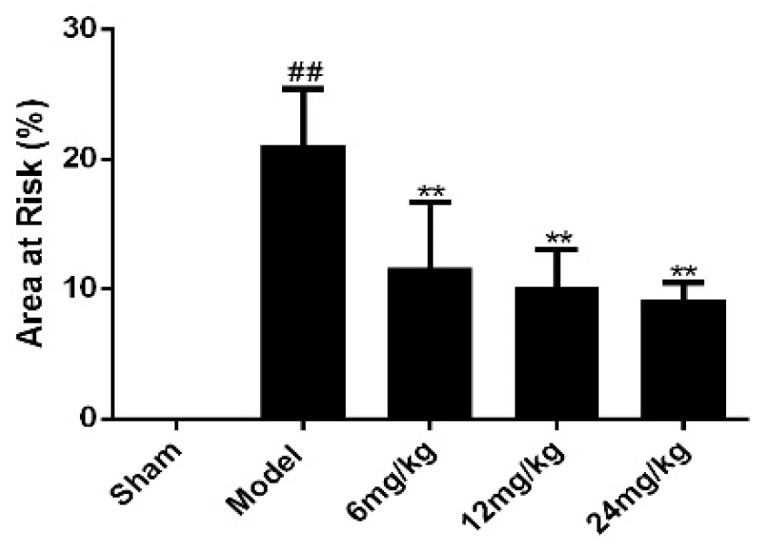
Sal B significantly reduced the infarct area of rats (*n* = 6 rats). TTC staining was used to evaluate the myocardial infarct size of each rat. Data were expressed as mean ± SD. ** *p* < 0.01 vs. Model group, ^##^ *p* < 0.01 vs. Sham group.

There was no obvious infarction in sham group, as showed in Figure 1. However, the infarct size of the model group was significantly higher than that of the sham group. Meanwhile, the infarct size of Sal B treatment groups was markedly lower than that of model group.

### 2.2. Effect of Sal B on the Electrocardiograph Parameters

**Figure 2:**
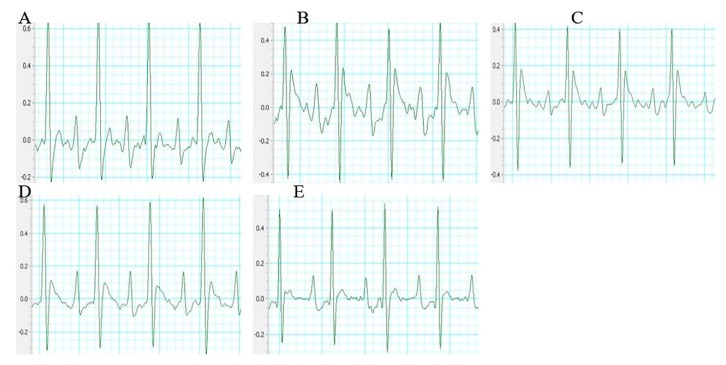
Representative electrocardiogram of each group (*n* = 10 rats). (**A**) Sham group (**B**) Model group (**C**) Sal B (6 mg/kg) (**D**) Sal B (12 mg/kg) (**E**) Sal B (24 mg/kg).

The electrocardiogram (ECG) patterns of each group rats were shown in Figure 2. Compared with the sham group rats, the ST segment in the model group rats were significantly higher. However, these changes were dramatically improved by the treatment with Sal B.

### 2.3. Sal B Alleviated the Pathological Changes of Rat Hearts

**Figure 3:**
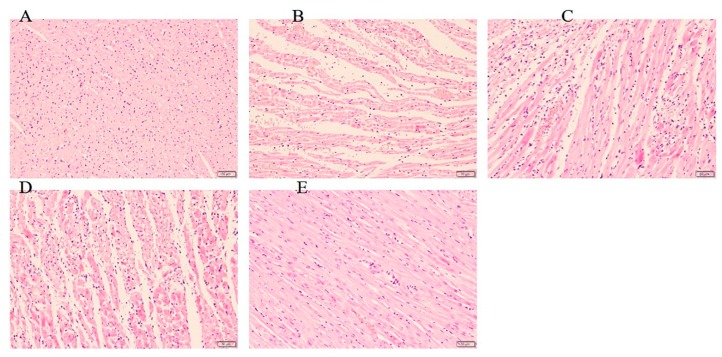
Histopathological observation of rat heart in each group (*n* = 3 rats). (**A**) Sham group, the myocardial fibers are arranged in an orderly manner. (**B**) Model group, myocardial fibers are partially ruptured and lysed, following significant inflammatory cell infiltration. (**C**) Sal B (6 mg/kg), (**D**) Sal B (12 mg/kg), myocardial fibers are partially ruptured and lysed, following moderate inflammatory cell infiltration. (**E**) Sal B (24 mg/kg) The cardiac fibrous rupture and inflammatory cell infiltration were significantly alleviated. (magnification ×200).

The slides of histologic pathology demonstrated that the hearts of rats in sham group maintained normal structure and shape. Besides, the myocardium injury and inflammatory cells infiltration in Sal B treated group were significantly less severe than did those in the model group (Figure 3).

### 2.4. Effects of Sal B on LDH/cTn/IL-1β in Serum of Myocardial Ischemia Rats and Cell Supernatant of H9C2 Cells

**Figure 4:**
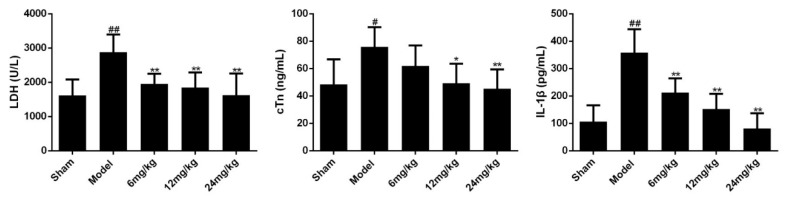
Effects of Sal B on LDH/cTn/IL-1β in serum (*n* = 6 rats). Rats were intravenous injected Sal B after coronary artery ligation. Data were expressed as mean ± SD. * *p* < 0.05, ** *p* < 0.01 vs. Model group, ^#^ *p* < 0.05, ^##^ *p* < 0.01 vs. Sham group.

The elevation of cardiac markers (such as LDH, cTn) and inflammatory cytokines (such as IL-1β) are important bases for the diagnosis of myocardial ischemia injury. To evaluate the efficacy of Sal B on myocardial ischemia, the expression levels of LDH, cTn and IL-1β in serum were determined. Results showed that myocardial ischemia resulted in significant increases in the levels of LDH, cTn and IL-1β (Figure 4). However, treatment with Sal B (6, 12, 24 mg/kg) remarkably alleviated these conditions.

**Figure 5:**
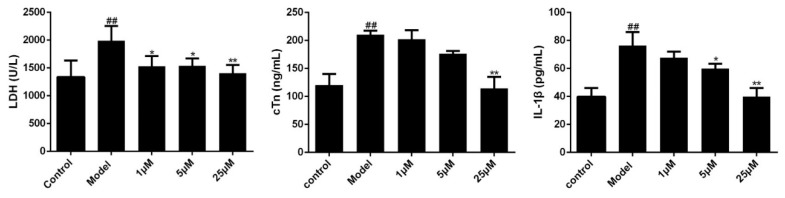
Effects of Sal B on LDH/cTn/IL-1β in cell supernatant (*n* = 3). Data were expressed as mean ± SD. * *p* < 0.05, ** *p* < 0.01 vs. Model group, ^##^ *p* < 0.01 vs. Control group.

Next, we examined these cytokines in H9C2 cell supernatant. And results showed that LPS stimulation significantly increased the expression levels of LDH, cTn and IL-1β (Figure 5). However, Sal B treatment (1, 5, 25 μM) notably reduced the expression levels of these cytokines.

### 2.5. Effects of Sal B on TLR4/NF-κB Signaling-Related mRNA Expressions in LPS-Induced H9C2 Cells

**Figure 6:**
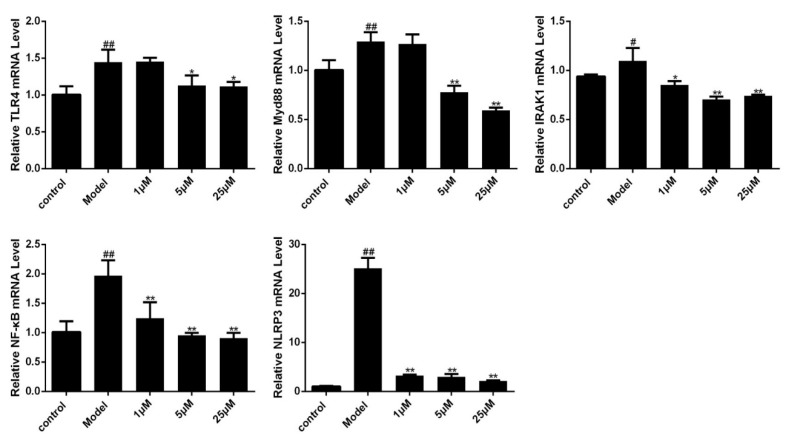
Effects of Sal B on TLR4/Myd88/IRAK1/NF-κB/NLRP3 mRNA levels in H9C2 as detected by fluorescence quantitative PCR (*n* = 3). Data were expressed as mean ± SD. * *p* < 0.05, ** *p* < 0.01 vs. Model group, ^#^ *p* < 0.05, ^##^ *p* < 0.01 vs. Control group.

To evaluate whether Sal B can reduce the NLRP3 inflammasome expression by inhibiting the priming phase, qPCR was used to examine the expression of related mRNA in TLR4/NF-κB signaling pathway. As shown in Figure 6, TLR4, Myd88, IRAK1, NF-κB, NLRP3 mRNA levels in the Sal B treated groups were significantly lower than those of the model group.

### 2.6. Effects of Sal B on TLR4/NF-κB Signaling-Related Protein Expressions in LPS-Induced H9C2 Cells

**Figure 7:**
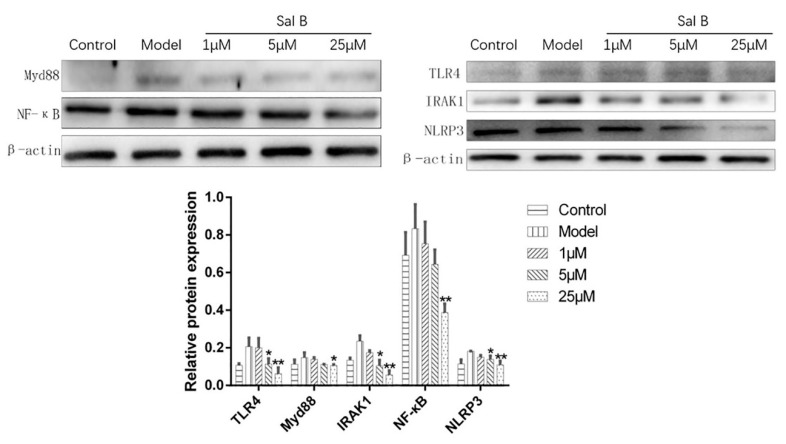
Effects of Sal B on TLR4/Myd88/IRAK1/NF-κB/NLRP3 protein expression levels in each group (*n* = 3). Expression was normalized to β-actin, data were expressed as mean ± SD of three independent experiments (*n* = 3). * *p* < 0.05, ** *p* < 0.01 vs. Model group.

**Figure 8:**
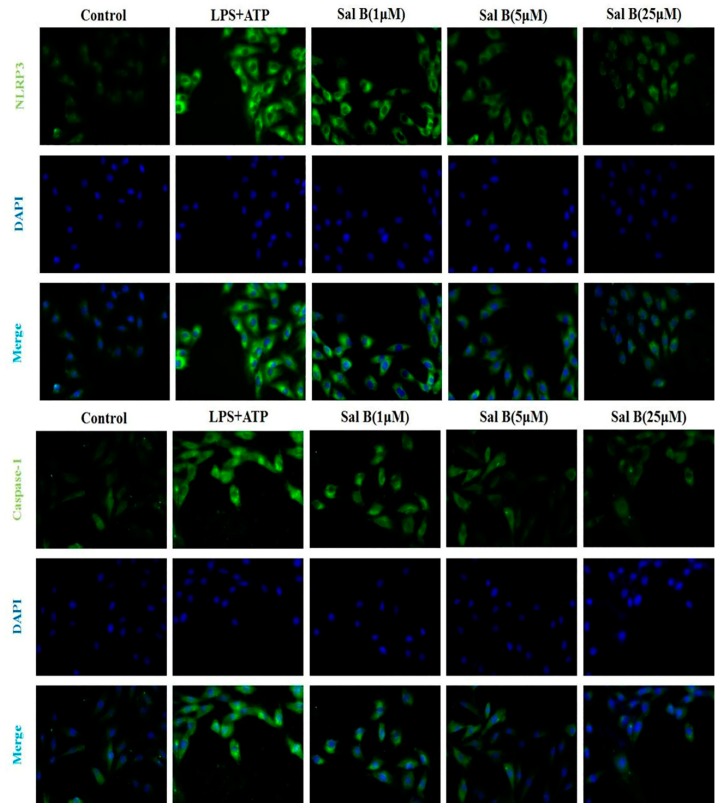
The expression levels of NLRP3 and caspase-1 were notable decreased in Sal B treated groups (*n* = 3).

To explore the underlying mechanisms of Sal B-mediated cardio protection, the protein expressions of TLR4/NF-κB signaling pathway were detected. Results showed that the protein expression levels of TLR4/Myd88/IRAK1/NF-κB/NLRP3 were significantly increased after modelling compared with the control group. However, the expression levels of these proteins were significantly reduced when treated with Sal B (Figure 7). Furthermore, the results of immunofluorescence also showed that Sal B could significantly reduce the expression levels of NLRP3 and caspase-1 in H9C2 after treated with LPS (Figure 8).

### 2.7. Docking Sal B to MD-2 Revealed the Binding Affinity

**Figure 9:**
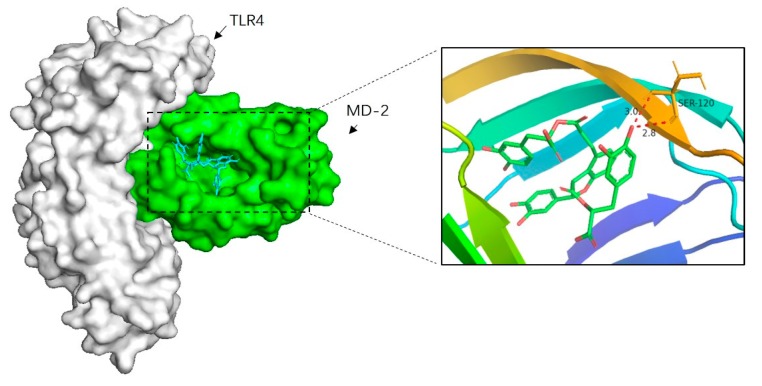
Molecular docking analysis of Sal B to the binding site of MD-2. Among the 50 output docking poses, this binding pose were indicated to have the highest docking score, and it formed strong interactions with hydrophobic residues at PHE-76, ILE-94, PHE-104, ILE-63, LEU-61, PHE-147, VAL-135, LEU-149, PHE-151, ILE-46, VAL-48, PHE-119, SER-120, ILE-52.

To further elucidate the mechanism of Sal B inhibiting TLR4/NF-κB signaling pathway, the molecular docking analysis of Sal B and MD-2 was performed. According to our present knowledge, LPS, HMGB1 and many other substances could bind to MD-2, which in turn leads to the dimerization of TLR4 and activates a series of inflammatory responses. Besides, the docking result showed that Sal B was fitted into the hydrophobic pocket of MD-2 and displaying close interaction with MD-2. Among the 50 output docking poses, this binding conformation (Figure 9) was indicated to have the highest docking score (−8.2 kcal/mol). In this binding mode, Sal B formed strong interactions with hydrophobic residues at PHE-76, ILE-94, PHE-104, ILE-63, LEU-61, PHE-147, VAL-135, LEU-149, PHE-151, ILE-46, VAL-48, PHE-119, ILE-52 and two hydrogen bonds with SER120. These interactions helped Sal B anchor in the binding site of MD-2, which suggest that it may inhibit the activation of inflammatory cascade through TLR4 and its relative signaling pathway.

### 2.8. Sal B Is a Specific MD-2 Inhibitor

**Figure 10:**
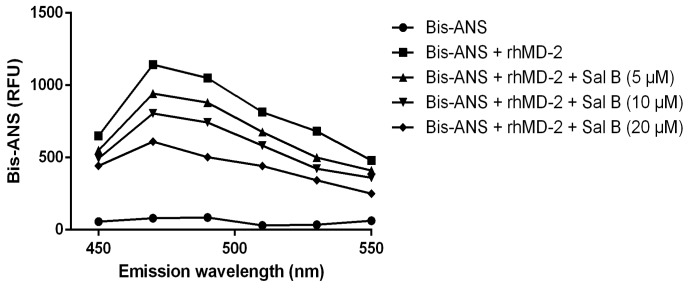
Fluorescence measurements showed that Sal B dose-dependently inhibited the binding of bis-ANS and rhMD-2.

The result showed that the fluorescence values of bis-ANS were markedly enhanced upon binding to the rhMD-2 protein, while incubation with Sal B decreased the fluorescence intensity in a dose-dependent manner (Figure 10), suggesting that Sal B may bind to the rhMD-2 protein directly.

### 2.9. Sal B Blocked LPS-Induced MD-2/TLR4 Association

**Figure 11:**
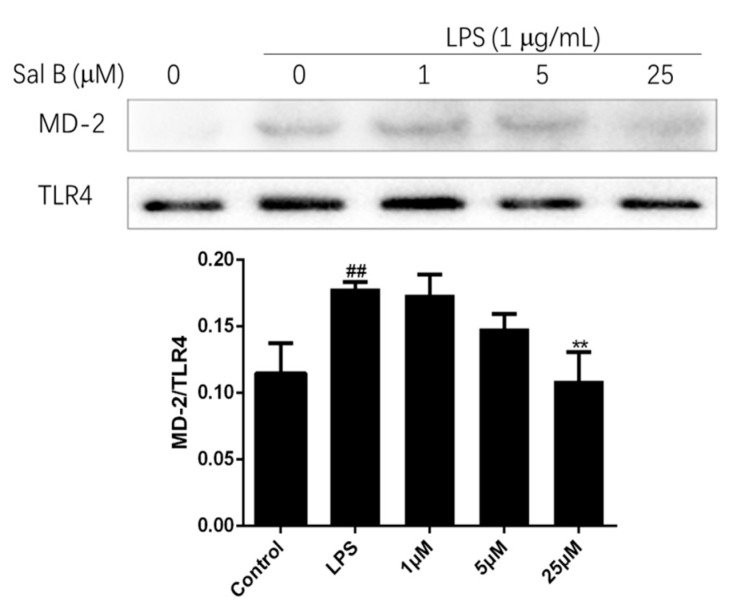
Sal B blocks LPS-induced MD-2/TLR4 association. H9C2 were pretreated with Sal B (0, 1, 5, 25 μM) for 24 h and then added LPS (final concentration: 1 µg/mL) stimulated for 24 h. After that, the expression levels of TLR4 and MD-2 were detected by immunoprecipitation and immunoblot. Densitometric analysis of the MD-2/TLR4 ratio in the immunoblots of the immunoprecipitates was carried out. Data were expressed as mean ± SD of three independent experiments (*n* = 3). ** *p* < 0.01 vs. LPS group. ##*p* < 0.01 vs. Control group.

To evaluate the affinity of Sal B to MD-2, the immunoprecipitation assay was performed. Briefly, the cell lysate of H9C2 was treated with anti-TLR4 antibody, and the expression levels of MD-2 and TLR4 were detect by immunoprecipitation and immunoblot. The result was shown in Figure 11, LPS treatment significantly increased the co-precipitation of TLR4/MD-2 complex, but Sal B significantly reduced the expression level of it in a dose-dependent manner. These results indicated that Sal B may inhibit the activation of TLR4/NF-kB signaling pathway through binding to MD-2.

## 3. Materials and Methods

### 3.1. Materials

Salvianolic acid B (Sal B) was provided by Nanjing Hongqiao Institute of Pharmaceutical Technology. Dulbecco’s modified eagle medium (DMEM) was purchased from KeyGEN BioTECH Co., Ltd. (Nanjing, China). Fetal bovine serum was purchased from Biological Industries Co., Ltd. (Israel). Trypsin was purchased from Beyotime Institute of Biotechnology Co., Ltd. (Shanghai, China). LDH assay kit was purchased from Nanjing Jiancheng Bioengineering Institute (Nanjing, China). IL-1β ELISA kit was purchased from multi sciences Co., Ltd. (Hangzhou, China). cTn ELISA kit was purchased from YIFEIXUE BIO TECH (Nanjing, China). Evagreen 2x qPCR MasterMix-Low Rox kit was purchased from Abmgoodchina Inc (Zhenjiang, China). Lipopolysaccharide (LPS) was purchased from Cell Signaling Technology, Inc (USA). Adenosine triphosphate disodium (ATP) was purchased from Sinopharm Chemical Reagent Co., Ltd. (Shanghai, China). Primary antibodies against Myd88/IRAK1/NF-κB/NLRP3/β-actin were obtained from proteintech group, Inc (Wuhan, China). Primary antibodies against MD-2 were obtained from Bioss Biotechnology Co., Ltd. (Beijing, China). Antibody for TLR4 was obtained from Santa Cruz Biotechnology (USA). Water used in this study was purified by a Milli-Q system (Millipore, MA, USA). All culture plates were obtained from corning (Corning, USA).

### 3.2. Animals

Adult male Sprague-Dawley rats (220–250 g) were purchased from Qinglong Mountain Animal Breeding Farm (Nanjing, China). The rats were provided with standard rat food and tap water under standard laboratory condition. This study was carried out in accordance with the principles of the Basel Declaration and recommendations of the Guidelines of Jiangsu Regulation for the Administration of Laboratory Animals. The protocol was approved by the Animal Ethics Committee of Nanjing University of Chinese Medicine (No. 201906A036). 

### 3.3. Experimental Protocol and Drug Administration

Sprague-Dawley rats were randomly divided into five groups (*n* = 10 rats), including the sham group, ischemia group, ischemia group + Sal B (6 mg/kg), ischemia group + Sal B (12 mg/kg), ischemia group + Sal B (24 mg/kg). Myocardial ischemic injury animal model was constructed by LAD ligation for 24 h as previous described [17]. In brief, rats were anesthetized with 3% sodium pentobarbital. The chest was opened through a left thoracic incision. A 6–0 silk suture slipknot was placed at the distal 1/3 of the left anterior descending artery. The electrocardiogram (ECG) of each rat was continuously recorded. After ligation, Sal B was injected into the tail intravenous of rat immediately. And the sham group was given the same dose of normal saline. 24 h later, all rats were sacrificed. Then the serum was collected for a series of biochemical assays. And the hearts were harvested for morphological, biochemical studies.

### 3.4. Measurement of Myocardial Infract Size

24 h after surgical ligation, all rats were sacrificed. 6 rats were randomly selected from each group to measure the myocardial infarct size using 2% 2,3,5-triphenyltetrazolium chloride (TTC) staining. The myocardial area at risk (AAR) were detected with a scanner, and data were analyzed with Image J. The red area was no infarct area, and the white or pale area indicated myocardial infarction, then the area of risk was calculated as follow:

$$\mathrm{Area}\;\mathrm{at}\;\mathrm{risk}\;\left(\%\right)=\frac{\mathrm{infract}\;\mathrm{area}}{\mathrm{whole}\;\mathrm{heart}\;\mathrm{area}}\times 100\%.$$

### 3.5. Histopathologic Observation

The hearts were immersed in 10% formaldehyde solution immediately and embedded in paraffin. Then the heart tissue was cut into 4 μm-thick sections. Tissue sections were stained with hematoxylin and eosin for observing the histopathologic changes under microscope.

### 3.6. Determinations of LDH, cTn and IL-1β in Serum

Blood was obtained from the abdominal aorta in each rat at 24 h after surgery. Then, serum LDH, cTn, IL-1β levels were assessed by a Microplate System according to the manufacturer’s instruction. 

### 3.7. Cell Culture and Treatment

H9C2 cells were obtained from American Type Culture Collection and routinely cultured in high glucose DMEM, supplemented with 10% FBS and incubated at 37 °C with 5% CO_2_.

### 3.8. Detections of LDH, cTn and IL-1β in Cell Supernatant

H9C2 cells were seeded in a 6-well plate at a density of 5 × 10^4^ cells. After overnight culture, the cells were pretreated with different concentrations of Sal B (1 µM, 5 µM, 25 µM) for 24 h. And the cell supernatant was discarded. The cell was washed twice with PBS. Then added LPS (final concentration: 1 µg/mL) stimulated for 24 h. After that ATP (final concentration: 5 mM) was added to stimulate for 2 h. The supernatant of each group was collected for analysis.

### 3.9. Immunofluorescence

H9C2 cells seeded on glass coverslips in 24-well plates were washed three times with PBS after treatments, then fixed for 30 min with 4% paraformaldehyde. After that washed cells twice with PBS, incubated with blocking buffer (1% BSA) for 1 h and subsequently incubated with primary rabbit polyclonal NLRP3/Caspase-1 antibody in blocking buffer for 12 h at 4 °C. Then washed the cells twice with PBS and incubated with DAPI goat anti-rabbit for 2 h at 37 °C. The images were acquired using a fluorescence microscope.

### 3.10. Quantitative Real-Time PCR

Total RNA was extracted from H9C2 cells using a Trizol reagent (Ambion, Waltham, MA, USA) according to the manufacturer protocols. The concentration of RNA was quantified using a Nanodrop (thermofisher, Waltham, MA, USA) and then subjected to reverse transcription using a cDNA reverse transcription kit (Abm, Zhenjiang) according to the instructions supplied by manufacturer. The relative quantification of these genes was carried out with an ABI 7500 Fast real-time PCR instrument (Applied Biosystems, Waltham, MA, USA) by using Evagreen 2x qPCR MasterMix-Low Rox kit with the following procedures: 95 °C for 10 min, followed by 40 cycles of 95 °C for 15 s, and 60 °C for 60 s. The relative mRNA amounts of TLR4, Myd88, IRAK1, NF-κB, NLRP3 were then calculated by comparative Ct method after normalizing against the quantity of GAPDH. The sequences of the primers were designed as follows (Table 1):

### 3.11. Western Blotting 

Total protein samples obtained from H9C2 cells were separated by 6%–10% SDS-PAGE and electro-transferred onto PVDF membranes. Then the membranes were blocked with 5% BSA for 1 h at room temperature and incubated overnight at 4 °C with primary antibodies against TLR4, MyD88, IRAK1, NF-κB p65, NLRP3 or β-actin. After washing three times with TBST, the membranes were incubated with secondary antibodies conjugated with horseradish peroxidase in non-fat milk for 1 h. Target proteins were detected with a gel electrophoresis apparatus (Bio-rad, Hercules, CA, USA), and visualized with the Image Lab.

### 3.12. In Silico Docking Simulations

The software used in silico assay were MGL Tools 1.5.6, Autodock Vina 1.1.2 [18] and pymol. The MD-2 structure (PDBID: 2E56) was taken from the protein data bank. pymol was used to remove the water and ligand structure in the protein. The MD-2 protein was further processed using MGL Tools 1.5.6 by adding polar hydrogen. And energy minimization. The 3D-structure of Sal B were drawn using ChemDraw 8.0 and energy minimized by computing Gasteiger and Marsili atomic charge method with MGL Tools 1.5.6. The prepared proteins and the ligands were then subjected to molecular docking using the Autodock Vina 1.1.2. The most favorable binding mode of Sal B was presented.

### 3.13. Bis-ANS Displacement Assay

Bis-ANS (Sigma, St. Louis, USA, 5 μM) and rhMD2 (5 nM) were mixed in PBS and allowed to reach stable fluorescence under excitation at 385 nm. Then different concentrations of Sal B (5 µM, 10 µM, 20 µM) were added, and relative fluorescence units emitted at 450–550 nm were measured. Fluorescence measurements were performed with a PerkinElmer EnSpire multimode reader.

### 3.14. Immunoprecipitation

A protein G immunoprecipitation kit (Sangon Biotech, Shanghai, China) was used for immunoprecipitation according to the manufacturer’s protocol. for 2 h at 4 °C 250 µg cellular protein was incubated with 2 µg anti-TLR4 antibody, followed by immunoprecipitation with 18 µL protein G beads overnight. Then the immunoprecipitates were washed 3 times with 700 µL IP buffer and subjected to immunoblotting with anti-MD2 antibody and anti-TLR4 antibody, respectively.

### 3.15. Statistics

All statistical analyses were conducted with GraphPad Prism 6.0, and the data were presented as mean ± SD. One-way ANOVA was used for multiple-group comparison. Differences were considered statistically significant when *p* < 0.05 (* *p* < 0.05, ** *p* < 0.01).

## 4. Discussion

In this paper, we found that Sal B could significantly alleviate myocardial ischemic injury and reduce the expression levels of many cardiac markers and inflammatory cytokines in serum, such as LDH/cTn/IL-1β. In addition, we demonstrated for the first time that Sal B could inhibit the activation of the TLR4/NF-κB/NLRP3 signaling pathway, and reduced the inflammatory cascade, thereby alleviating myocardial ischemic injury.

Although the underlying mechanisms of myocardial ischemia are still not fully understood, many studies have shown that myocardial ischemia is often accompanied by severe inflammation. When the body is stimulated by external stimuli, the innate immune system is first activated [19]. Subsequently with the induction of TLR4 expression, the adaptive immune response was activated. NF-κB is then activated through the MyD88-dependent signal pathway. Some endogenous ligands could activate NF-κB through the activation of TLR4 receptor, causing the cascade of inflammatory reactions [20]. As a component of innate immunity [21], the NLRP3 inflammasome plays an important role in many inflammatory diseases [22,23]. Our research showed that Sal B could inhibit the activation of NF-κB signaling pathway and the expression of NLRP3 inflammasome in a dose-dependent manner in LPS induced H9C2 cells.

**Figure 12:**
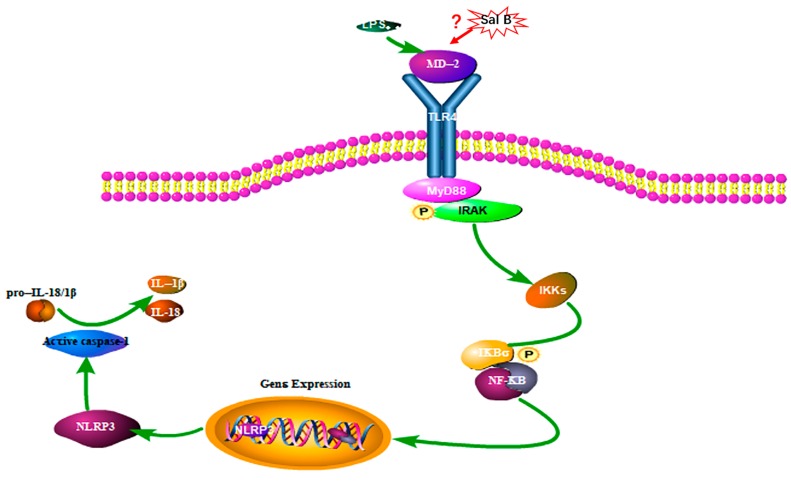
Pathway of Sal B on LPS induced H9C2 cells (P: Phosphorylation).

However, the dimerization of TLR4 was proven to be a prerequisite for the ligand-induced (such as LPS, HMGB1, et.) [24] TLR4 activation and acts upstream of MyD88/NF-κB signaling pathway [25]. To our current knowledge, external stimuli such as LPS or hypoxia could cause cell damage and release HMGB1. Then LPS or HMGB1 will bind to MD-2 and lead to the activation of TLR4/NF-κB signaling pathway. The molecular docking results showed that Sal B was embedded into the hydrophobic pocket of MD-2, which may indicate competing and overlapping with the binding sites of them. Therefore, the anti-inflammatory effect of Sal B may initially be due to its ability to block TLR4 dimerization. Simplified overview of the above signaling pathways was as illustrated in Figure 12.

## 5. Conclusions

Our study demonstrated that the activation of the TLR4/NF-κB/NLRP3 signaling pathway plays an important role in myocardial ischemic injury, which promotes the cascade of inflammatory responses and exacerbates myocardial ischemic injury. Sal B, as a natural product with little toxicity, can provide new prospects for the treatment of myocardial ischemia.

## Figures and Tables

**Table 1:** Oligonucleotides used for qPCR.

Oligonucleotide	Forward Sequence (5’–3’)	Reverse Sequence (5’–3’)
TLR4	GCTCTCAACCTTGGTACTGACAGG	GTCTCCACAGCCACCAGATTCTC
Myd88	CGACGCCTTCATCTGCTACTGC	CCACCACCATGCGACGACAC
IRAK1	GCGTGTGGCTGACCTCGTTC	GGAGAGGAAGGTGGAGGCAGAG
NF-κB	TGTGGTGGAGGACTTGCTGAGG	AGTGCTGCCTTGCTGTTCTTGAG
NLRP3	CTCTGCATGCCGTATCTGGT	GTCCTGAGCCATGGAAGCAA
GAPDH	GGCAAATTCAACGGCACAGT	AGATGGTGATGGGCTTCCC
